# Recognizing Epithelial Cells in Prostatic Glands Using Deep Learning

**DOI:** 10.3390/cells14100737

**Published:** 2025-05-18

**Authors:** Liton Devnath, Puneet Arora, Anita Carraro, Jagoda Korbelik, Mira Keyes, Gang Wang, Martial Guillaud, Calum MacAulay

**Affiliations:** 1 Integrative Oncology, BC Cancer Research Centre, Vancouver, BC V5Z 1L3, Canada; parora@bccrc.ca (P.A.); acarraro@bccrc.ca (A.C.); jkorbeli@bccrc.ca (J.K.); mguillau@bccrc.ca (M.G.); 2 Pathology and Laboratory Medicine, University of British Columbia, Vancouver, BC V6T 2B5, Canada; gang.wang1@bccancer.bc.ca; 3 BC Cancer Agency, Vancouver, BC V5Z 4E9, Canada; mkeyes@bccancer.bc.ca; 4 Radiation Oncology, University of British Columbia, Vancouver, BC V6T 1Z4, Canada

**Keywords:** prostate cancer, prostatic glands, stoichiometric DNA stain, epithelial cell, stromal cell, machine learning, semi-supervised method, deep learning

## Abstract

Artificial intelligence (AI) is becoming an integral part of pathological assessment and diagnostic procedures in modern pathology. As most prostate cancers (PCa) arise from glandular epithelial tissue, an AI-based methodology has been developed to recognize glandular epithelial nuclei in prostate biopsy tissue. An integrated machine-learning network, named GlandNet, was developed to correctly recognize the epithelial cells within prostate glands using cell-centric patches selected from the core biopsy specimens. Feulgen-Thionin (a DNA stoichiometric label) was used to stain biopsy sections (4–7 µm in thickness) from 82 active surveillance patients diagnosed with PCa. Images of these sections were human-annotated, and the resultant dataset consisted of 1,264,772 segmented, cell-centric nuclei patches, of which 449,879 were centered on epithelial gland nuclei from 110 needle biopsies (training set: *n* = 66; validation set: *n* = 22; and test set: *n* = 22). The training of GlandNet used semi-supervised machine-learning knowledge of the training and validation cohorts and integrated both human and AI predictions to enhance its performance on the test cohort. The performance was evaluated against a consensus deliberation from three observers. The GlandNet demonstrated an average accuracy, sensitivity, specificity, and F1-score of 94.1%, 95.7%, 87.8%, and 95.2%, respectively, when tested on the 20,735 glandular cells found in the three needle biopsies with the visually best consensus predictions. Conversely, the average accuracy, sensitivity, specificity, and F1-score were 90.9%, 86.4%, 94.0%, and 89.7% when assessed on 57,217 cells found in the three needle biopsies with the visually worst consensus predictions. GlandNet is a first-generation AI with an excellent ability to differentiate between epithelial and stromal nuclei in core biopsies from patients with early prostate cancer.

## 1. Introduction

Prostate cancer (PCa) is a leading cause of cancer-related deaths among males in North America. In 2024, cancer statistics in the United States projected that there were 299,010 new cases of prostate cancer identified in men, resulting in 34,130 deaths [1].

The development of cancer is a consequence of genetic alterations that lead to the accelerated replication of cells and loss of tissue homeostasis. There is a high probability that these genetic alterations are caused by a confluence of variables, including the patient’s initial genetic makeup and history of environmental exposure to carcinogens. Most prostate cancers originate from glandular epithelium where aberrant epigenetic and genetic modifications can lead to malignant transformation. Accurately identifying cell type and differentiating them into either epithelial or stromal origin is crucial for studying the role of the transformed epithelial cells and their microenvironment (TME) in tumor growth and progression [2,3].

In 1966, Donald Gleason developed a grading and scoring system for prostatic adenocarcinoma, and since 1974 it became the universally accepted. The Gleason classification system is most often used to grade prostate cancer, where histological glandular growth patterns (grades) of prostate adenocarcinoma are clinically correlated with staging, treatment recommendations, and ultimately disease prognosis. Unfortunately, the variability in Gleason grading can be significant between different observers, due to its somewhat subjective nature subject to expertise and opinions [4].

The number of epithelial cell and cell layers increase as a consequence of malignant transformation and deregulation of the glandular structure. The glands in a normal prostate are mostly made up of a single layer of glandular cells arranged on top of the myo-epithelial cells. Abnormal prostatic glands usually have glandular cells > 2 cell layers [5]. As the number of layers increases, cells move farther away from the basal layer. Prostate adenocarcinomas lose the basal myo-epithelial cells, with invasive proliferation of the neoplastic glandular cells. Gleason grading patterns are based on tumor gland architecture and the glandular relationship to stromal matrix [6].

With varying degrees of success, many studies have used systems biology and machine-learning approaches to correlate pathology between human observers (pathologists) and AI [7,8,9]. The biology technique, which involves quantifying the expression and pattern of several molecular markers, has been linked to aggressive behavior and prostate cancer. Recent hematoxylin and eosin (H&E) stained-based studies have demonstrated that deep learning-based algorithms can perform Gleason grading with an equivalent level of accuracy as diagnoses provided by experts [10,11,12,13,14]. There are a few artificial intelligence (AI)-driven glands-based studies that have used the H&E stain to classify benign and cancerous glands without specifying the involvement of cells [15,16,17].

As cancer is a disease of the genetic material within cells, it has been shown that quantifying the amount and organization of DNA within cells labelled with a stoichiometric DNA stain and using DNA ploidy and DNA organization within nuclei can be used to recognize aggressive cells within prostate glands and other tissue sites [5,6,18]. While the methodology of these studies can be high throughput, the exception is when the annotation of glandular cells is necessary. Developing an automated prostate glandular cell recognition tool could aid in the recognition of aggressive cells within prostate glands, improving tools based upon DNA specificity for the prediction and prognosis of prostate cancer. The objective of this study was to develop a novel system that could correctly differentiate between glandular epithelial cells and stromal cells in the prostate for DNA-specific stained cells.

As cancer is a disease in the inheritable characteristics of cells (genetic and epigenetic DNA structure and organization), we and others have utilized a stoichiometric DNA stain (Feulgen-Thionin) to label the DNA of the cells and used the quantification of the DNA at each pixel and how it is distributed within the pixels that make up the nuclei to predict biological aggressiveness of the tissue under study [5,19]. Thus, for this work we are working with Feulgen-Thionin-stained tissues.

**Figure 1:**
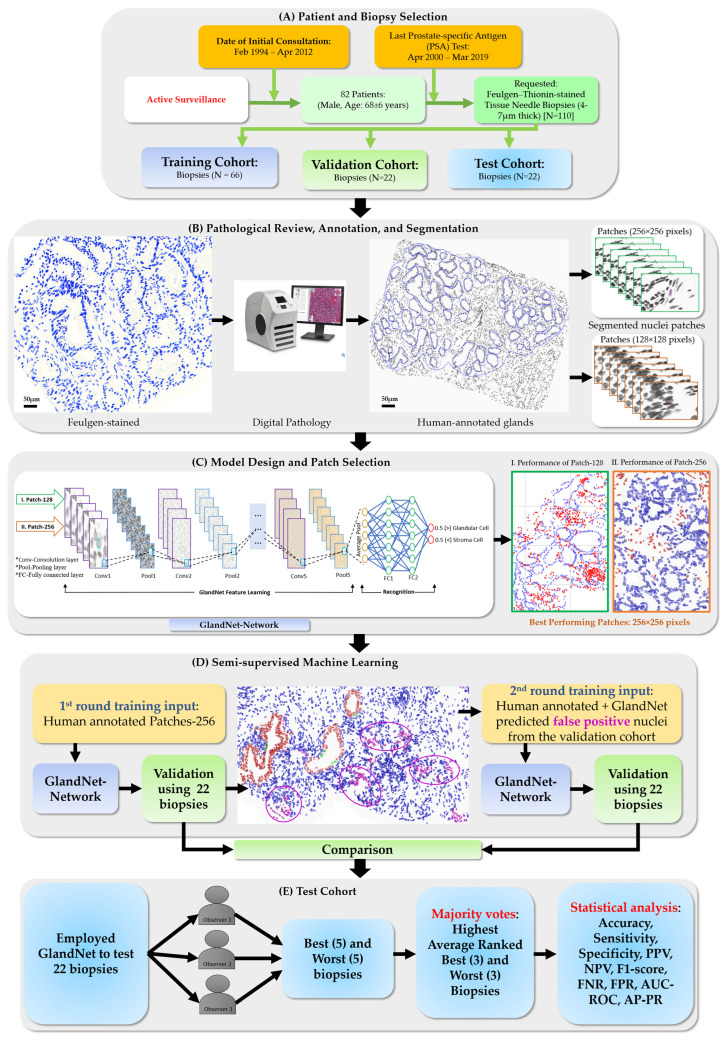
Integrating machine learning into glandular cell recognition workflow. The method has multiple steps: (**A**) patient and biopsy selection (110 Feulgen-Thionin-stained needle biopsies from 82 patients were organized into three cohorts for this experimental study); (**B**) pathological review, annotation, and segmentation, showing annotated glandular structures (blue lines) in Feulgen-stain biopsies (the biopsies are then segmented, and either a 128-by-128 or 256-by-256 batch is centered on each segmented nuclei); (**C**) model design and patch selection (in a detailed analysis of CNN models utilizing the two sizes of patches (Patch-128 and Patch-256), Patch-256 patches had superior performance); (**D**) semi-supervised machine learning, resulting in an expansion and recursive modification of the GlandNet training set, integrating the false-positive data from the 1^st^ round predictions as true positives in the 2^nd^ round to enhance generalization; (**E**) test cohort, where cells from 22 biopsies were predicted using the trained model from the 2^nd^ round. Three observers were selected and ranked the biopsies to find the visually 5 best and 5 worst predictions. The intersection of all 3 observers’ selections identified the biopsies with the 3 best and 3 worst predictions. For these 6 biopsies, an annotator classified all the individual identified cells as gland or non-gland cells, and these annotations were utilized to assess the performance of GlandNet.

Our research group developed a technique for segmenting the nuclei of Thionin-stained prostate needle biopsies [18,20]. In the first part of this pilot project, this model was used to generate cell-centric patches from our manually annotated prostate needle biopsies. What was developed in this study was an integrated machine-learning network, GlandNet, to automate the recognition of segmented nuclei that are part of a prostatic glands and those that are not. Further, a semi-supervised machine-learning technique was used to improve the model’s generalization [21,22,23,24]. The subsequent model and its (GlandNet) improvements, progressing from training, validation, and the test cohorts of biopsies (Figure 1A), were evaluated. The results were assessed by three observers. What was assembled was a dataset of Thionin-stained prostate needle biopsies that had their glandular structures manually annotated as part of a previous study. The nuclei in the needle biopsies were segmented using a sequential UNet process [20], and these segmented nuclei were used to generate two different patch sizes: Patch-128 (128-by-128 pixels) and Patch-256 (256 by 256), where every patch is centered on a segmented nucleus. The patch data was divided into training, test, and validation sets at the biopsy level. The study approach was as follows:

- For each size of patches, the accuracy of the trained GlandNet was investigated to determine the effect of patch size on prediction performance, and we identified the one with superior performance.
- GlandNet development was continued using the best-performing patch size (Patch-256) and a semi-supervised approach conducted using the training and validation datasets.
- In the second-round training, GlandNet integrated both human and machine predictions to boost its accuracy, and then it was applied to the test cohort.
- Prostate glandular cell recognition in the test cohort was evaluated through a majority vote process among three observers to visually identify the three biopsies with the best predictions and the three biopsies with the worst predictions. For these six biopsies, all the 77,952 cells were hand-annotated (C.M.) as within glands or not within glands to estimate the accuracy range of GlandNet.

Figure 1 illustrates an overview of an integrated machine-learning network for detecting prostatic glands using cell-centric nuclei patches from prostate needle biopsies. The manuscript is structured as follows: a summary of data (Section 2.1), review and annotation (Section 2.2), and a nuclei segmentation approach (Section 2.3) under the following materials and methods in Section 2. The details about GlandNet (Section 2.4) and its implementation strategies (Section 2.5) are also discussed separately under the Section 2. The explanation of the results in Section 3 discusses the visual assessment of GlandNet’s predictions for each cohort: training (Section 3.1), validation (Section 3.2), and test (Section 3.3) independently. The discussion in Section 4 discusses the overall results of GlandNet, its contributions, limitations, and potential challenges. We concluded our overall findings and future work in the conclusion (Section 5) of the study.

## 2. Materials and Methods

### 2.1. Sample Preparation

In adherence to the ethical guidelines set forth by the University of British Columbia and BC Cancer Research Ethics Board (ethics approval certificate numbers H13-01398), tissue needle biopsy blocks from 82 patients (male; age: 68 ± 6 years; tissue type: B) diagnosed with prostate cancer (Gleason 6 or 7) who were in active surveillance were requested, and 4–7 µm thick sections were cut from each block. One section was stained with H&E to determine which needle biopsies still contained diagnostic tissue. In those needles biopsied, it was confirmed to contain diagnostic tissue, and the immediately adjacent section was stained using a Feulgen-Thionin stain with DNA-specific absorption that was stoichiometric for DNA [18]. As this study involved the secondary use of data, only a waiver of consent was granted by the Research Ethics Boards at UBC-BC Cancer, and consent was not required from the patients from which the biopsies were collected.

### 2.2. Pathological Review and Annotation

**Figure 2:**
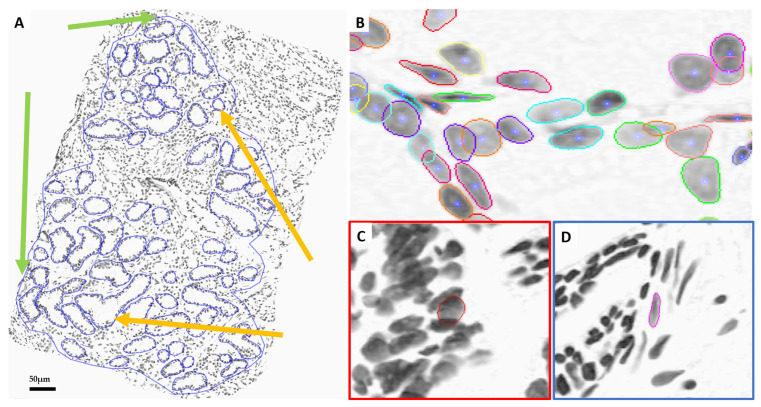
(**A**) Human-annotated Thionin-stained needle biopsy (pathologist-annotated large ROI: green arrow; annotated glands: orange arrow); (**B**) sequential UNet segmented nuclei, in which small blue dots are nuclei centers and colored contours are segmented nuclei boundaries; (**C**) segmented nuclei at center of Patch-128 (middle red box); (**D**) segmented nuclei at center of Patch-256 (right blue box).

Digital scanning of the slides was performed using a whole slide panorama MIDI scanner (3DHISTECH) that used a color CCD camera and a 20 × 0.8 NA lens. Pixel spacing was 0.329 µm in the sample plane for this system. The Region of Interest (ROI) that was designated by the pathologist on the H&E-stained adjacent slide and the equivalent area on the Thionin-stained slide was annotated. Two expert cyto-technicians were responsible for interactively delineating all glandular cells on the adjacent 82 patients’ Thionin-stained sections (see Figure 2A).

### 2.3. Nuclei Segmentation

A sequential UNet trained to segment Thionin-stained nuclei was used to find all the centers and boundaries of the nuclei in the Thionin-stained needle biopsies [18]. The coordinates of the detected centers were saved to generate new patches/tiles with each of the identified nuclei arranged in the center of the patch. The blue dots and other colors lines in Figure 2B indicate the centers and boundaries of the segmented nuclei. Each segmented cell-centric nuclei patch for a needle biopsy was saved in an in-house file format (CMG) which consisted of the image patch and its corresponding nuclear boundary and the spatial location of the patch in the larger image for every nucleus, all appended together in a single file per biopsy, which can be used to reconstruct the original biopsy image with segmentation boundaries. We generated two sizes of nuclei-centric patches (128-by-128 and 256-by-256 pixels) as shown in Figure 2C,D to investigate which patch size would generate the most accurate predictions while requiring the least computational resources.

### 2.4. GlandNet Algorithm Design

GlandNet is a 16-layer convolutional neural network (CNN) based upon the VGG16 network [25,26]. In order to accomplish the objective of distinguishing glandular cells from stromal cells, we used a Softmax (1) function as part of the final prediction layer [27]. The input to GlandNet was based upon the use of segmented nuclei to generate cell-centric images that had one of the segmented nuclei in its center (so a cell-centric image or patch) to generate probabilities that each nucleus was either in the stroma or in a prostate gland. During the training and validation of the GlandNet algorithm, we used the stochastic optimizer Adam along with the weighted categorical cross-entropy (CE) loss (2):

(1) $$Softmax(y_{probability_{i}})\;=\;\frac{\exp\left(y_{i}\right)}{\sum_{j\;=\;1}^{N\;=\;nClass}\exp\left(y_{i}\right)}$$

(2) $$CE\;=\;-\overset{N\;=\;nClass}{\underset{i\;=\;1}{\sum }}y_{truelabel_{i}}\times \log\left(Softmax(y_{probability_{i}})\right)\;=\;-\overset{N}{\underset{i\;=1}{\sum }}y_{i}\times \log\left(\hat{y_{i}}\right)$$

This CNN network trained on our proposed prostate gland cell-centric dataset as depicted in Figure 1A. In GlandNet, there are thirteen convolutional layers, five max and one global average pooling layers, two Gaussian noise layers, and three Dense layers which sum up to 24 layers, but it has only sixteen weight layers, i.e., learnable parameter layers. As a result of GlandNet’s ability to enhance the flow of information and gradients across the network, the optimization of networks becomes more straightforward. After the feature learning in network, as shown in Figure 1C, we added a global average pooling layer, followed by a dense layer, a Gaussian noise layer, another dense layer, a second Gaussian noise layer, and a final dense layer, replacing all fully connected (FC) layers. If the output probability of the final layer is greater than 0.5, we classified it as a glandular cell; otherwise, it is recognized as a stromal cell. To make the neural network non-linear, we implemented a Softmax activation after the last layer.

The initial weights for GlandNet were derived from a pretrained ImageNet model (VGG16) [25]. We trained the network for prostatic gland data using the Adam optimizer with standard parameters (β1 = 0.9 and β2 = 0.999), including a very low initial learning rate (lr = 0.00001) [28]. In addition, the training data was augmented by randomly flipping half of the patches horizontally and vertically, rotating them by degrees ranging from 0 to 45, zooming within the patches by 20%, and translating the width by 20% vertically and the height by 20% horizontally. The implementation of this technique has already demonstrated effectiveness in other imaging applications [29,30]. By employing this augmentation technique, variants of the images are generated in order to enhance the fit models’ capacity to extrapolate acquired knowledge from novel images [31,32,33]. We optimized the training algorithm by tuning the various combinations of GlandNet parameters, augmentation factors, batch size (32 to 128), learning rate (10^−2^ to 10^−5^), training epoch (50–100), and standard deviation (0.5 to 3.5) of the noise distribution within the training data.

### 2.5. GlandNet Implementation with Semi-Supervised Learning Technique

Each CMG file contained multiple cell-centric patches of segmented nuclei. We utilized a total of 110 CMG files (1,264,772 nuclei with 449,879 in glands) of biopsies to develop the GlandNet algorithm for glandular cell recognition.

For the glandular cell recognition task, we randomly split the 110 biopsies at the case level into training (66 cases, including 646,426 nuclei with 213,702 in glands), validation (22 cases, including 210,982 nuclei with 60,310 in glands), and test (22 cases, including 407,364 nuclei with 175,867 in glands) cohorts. A two-round training strategy was executed. During the first training round, the GlandNet model was trained using the training dataset that had been annotated by humans. Three observers were employed to examine the model’s performance on the validation dataset after each round of training. In the validation cohort for the first round of training, we observed most of the human-annotated stroma cells that the first-round model predicted as glandular cell and thus labeled as false positives, which were, upon review, in fact in glands (predominately smaller ones) upon closer evaluation. Thus, we decided to train the second-round model with both labeled (human-annotated gland cells) and unlabeled (false positive cells from the first round) datasets as shown in Figure 1D. In the second round of training, we added the first-round-predicted false positive nuclei (annotated label was negative/stroma but first-round prediction was glandular cell) that were identified in the validation cohort into the training dataset. We used the weights from the semi-supervised learning model that demonstrated the most efficient training and validation accuracy vs. loss at the 100th epoch to predict glandular cells in the test biopsies. As the number of individual cells (407,364) in the test cohort was too large to feasibly annotate by hand, each of the 3 observers was given the task of independently ranking the top five and the worst five predicted biopsies so as to estimate the performance range of GlandNet. We required a consensus across three observers for the biopsies to be in the best top or worst group. We identified 3 biopsies that all the observers agreed were in the best 5 and 3 biopsies that all the observers agreed were in the worst 5 of the test set. The 77,952 cells in these 6 biopsies were hand-annotated, and the range of the performance of GlandNet in prostate glandular recognition was assessed statistically across these 6 biopsies. We compared the outcomes using the following statistical measures: accuracy, sensitivity, specificity, positive prediction value (PPV), negative prediction value (NPV), false negative rate (FNR), false positive rate (FPR), F1-score, area under the ROC curve (AUC), and area under the precision-recall (PR) curve (AP).

## 3. Results

### 3.1. Training Cohort: Patch Size Selection and Tuning the Process

**Figure 3:**
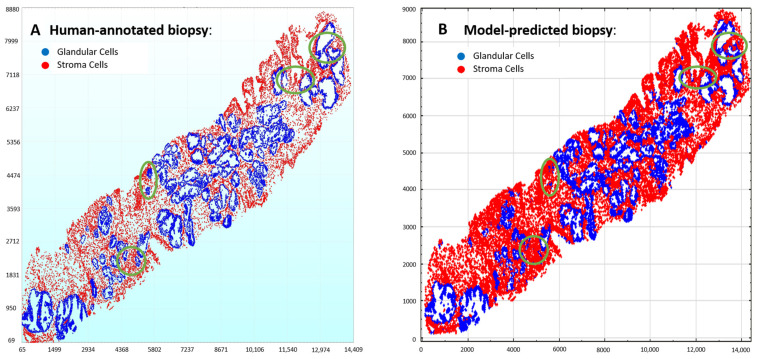
Results of GlandNet when trained using the 128-by-128 patches. (**A**) The results of the human-annotated biopsy are shown in the left image, while the prediction results are shown in the right image (**B**). According to human annotation, the mispredicted glands are highlighted by the green circles.

We investigated which cell-centric patch size is appropriate for our proposed GlandNet architecture. Initially, we trained the GlandNet network with the segmented cells centered in patches 128 by 128 in size. Table 1 demonstrates GlandNet’s overall performance across three random splits of the test dataset including the 41,022 hand-annotated glandular cells. We saw that, when we used 128-by-128 patches, the specificity (true negative rate) and NPV averages were both over 90%, while the sensitivity (true positive rate) and PPV were highly variable with an average of 58.9% (range 91 to 15.5%) for sensitivity and 72.1% (range 83.3 to 49.9%) for PPV. We analyzed the nuclei prediction mapping results of the GlandNet model, trained on the 128-by-128 patches (Figure 2C), as in Figure 3. In the human-annotated biopsy (Figure 3A), gland nuclei are blue, and stroma are red.

As our goal was to develop an integrated machine-learning model that would match human observation, these results did not meet our target. As demonstrated in Figure 3B, this instance of the algorithm struggled to identify glandular cells highlighted with a green circle that humans had annotated in Figure 3A. We speculated that this could be due to the limited image size of the patches. The patches (128 by 128) may not contain enough contextual information surrounding the cell-centric nucleus. We postulated that perhaps using a larger 256-by-256 patch potentially would enable the GlandNet learning approach to succeed. Therefore, we retrained our proposed model placing the segmented nuclei at the center of larger 256-by-256 patches with the gland status annotated as shown in Figure 2D.

**Figure 4:**
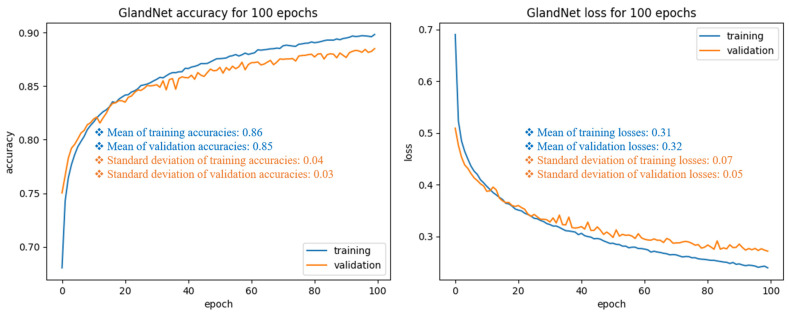
GlandNet’s training and validation performances throughout 100 epochs. Each plot also displayed the mean and standard deviation of the total 100 epochs of training and validation performance.

We optimized the training process by adjusting the GlandNet parameters, which include a few data augmentation aspects. The methodology section describes values we empirically found as sufficient for the data enhancement parameters. Our best training and validation performance was achieved with the batch size of 128, learning rate (10^−5^), and standard deviation of the added noise distribution (1–1.5). These settings enabled us to develop a well-suited learning algorithm by limiting the problems of overfitting and under-fitting. Figure 4 depicts the training and validation performance, including mean and standard deviation per epoch, in relation to loss and accuracy. In the initial step of training, GlandNet had a severe deficit in its ability to learn, as seen by the variability of the validation loss versus accuracy, which was unexpected when compared to the training. This occurred due to the poor parameter choices. When we chose parameters that were either higher or lower than the optimal, both the training and validation performance deteriorated. We did not use the GridSearch settings for our GlandNets due to their variability across different datasets [34,35,36,37]. As seen in the case in Figure 4, both the training and validation graphs exhibited a smooth improvement when the parameter setting were optimal. We picked the most proficient model for testing, which exhibited the best training and validation accuracy relative to loss for the final epoch.

### 3.2. Validation Cohort: Semi-Supervised Learning

The 256-by-256 patch-trained GlandNet predicted the patch status (and hence the status of the cell at the center of the patch) on the validation dataset. The biopsy prediction results for each biopsy were examined by three observers. The observers found differences between the human and AI/deep learning predictions for these biopsies for the first round of training. The reviewers observed that for most of the human-annotated stroma cells (annotated as negative), which the model identified as false positives (predicted to be in glands), were upon review, in fact, glandular cells which were mislabeled when the nuclei were being manually annotated. This likely occurred as it is very difficult for a human observer to differentiate between all the glandular nuclei in a complex prostate tissue region, and the annotation of the glands was a side product of the original analysis in which the biopsy images were originally collected for and not the focus of that study. As a result, the similarity between model prediction and the initial observer’s annotation was less than desired. To compensate for the double hit these mislabeled nuclei produced during training (to both the positive and negative prediction error terms during training), the 1^st^-round GlandNet model’s positive predictions were used to argument the human annotations. Therefore, in the second round, we integrated both human and machine predictions within gland nuclei as positive for retraining. We refer to this approach as “semi-supervised learning” and found that it enhances the generalizability of the model by combining human-annotated gland cells and the predicted to-be gland cells from the first round even though they were labeled as non-gland cells (false positives) by the annotators.

The second-round predictions for the test biopsies were assessed by three observers. Each observer ranked what they thought were the best and worst five biopsy prediction cases. The three biopsies that had the highest average ranking across the three observers were identified (Best 3), and the three biopsies that had the lowest average ranking across the three observers were identified (Worst 3). In both the best three and worst three biopsies scenarios, the model validation accuracy, specificity (true negative rate), and PPV (positive prediction value) of the model exhibited a decrease (Supplementary Table S1), while sensitivity (rate of true positives) and NPV (value of negative predictions) demonstrated an increase in the second round of training (Supplementary Table S2).

**Figure 5:**
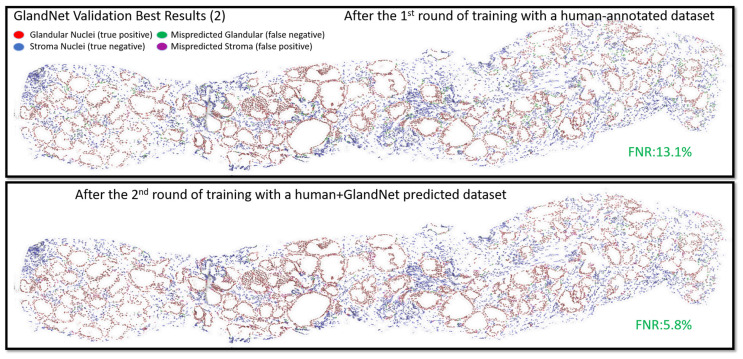
GlandNet validation results of one of the best-ranked biopsies after the 1^st^ and 2^nd^ evaluation rounds. Red nuclei are correctly identified glandular cells, blue nuclei are correctly identified stroma cells, green nuclei are missed glandular cells, and magenta nuclei are stroma cells incorrectly identified as glandular cells.

**Figure 6:**
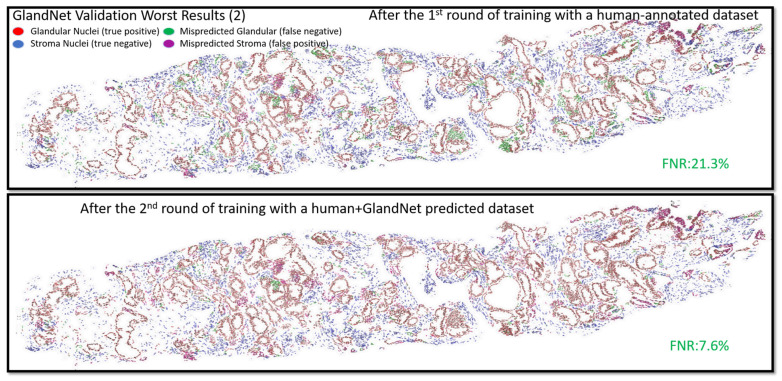
GlandNet validation results of one of the worst-ranked biopsies after the 1^st^ and 2^nd^ evaluation rounds. Red nuclei are correctly identified glandular cells, blue nuclei are correctly identified stroma cells, green nuclei are missed glandular cells, and magenta nuclei are stroma cells incorrectly identified as glandular cells.

Following the completion of the second round of training, the model’s generalization regarding glandular nuclei was improved by the semi-supervised learning approach. As a consequence, in both the best and worst validation results (2), the model significantly reduced the false negative rate (FNR) from 13.04% to 5.76% and 21.22% to 7.57%, respectively, as seen in Figure 5 and Figure 6. Supplementary Figures S1 and S2 illustrate validation results of the best-ranked (1 and 3), where the GlandNet model reduced the FNR from 14.64% to 7.76% and 15.54% to 8.18% after semi-supervised training. Additionally, the worst-ranked validation results (1 and 3) are shown in Supplementary Figures S3 and S4. These highlight how significantly the FNR drops, even with the worst scenarios. The training and validation cohorts were deemed satisfactory by three observers, prompting the decision to proceed with the test cohort.

### 3.3. Test Cohort: Majority Votes Across Three Observers

A total of 22 biopsies were used to test the GlandNet performance and the predictions evaluated by the three observers. The GlandNet weights from the 2^nd^ round of training were stored at the final epoch in order to make predictions on the test dataset. Each observer was assigned the responsibility of identifying the best five and the worst five biopsy results predicted by the GlandNet algorithm, as shown in Figure 1E. From an intersection of the three observers, the best three and most worst three biopsies were identified. For these six biopsies, an examiner (CM) examined the 77,952 cells and labeled each cell as either a gland cell or a non-gland cell, and these labels were used to evaluate how well GlandNet performed. The GlandNet was applied to the 20,735 glandular and 57,217 stroma cells and found that, for the worst three needle biopsies, the algorithm had an average accuracy, sensitivity, specificity, and F1-score of 90.9%, 86.4%, 94.0%, and 89.7%.

**Figure 7:**
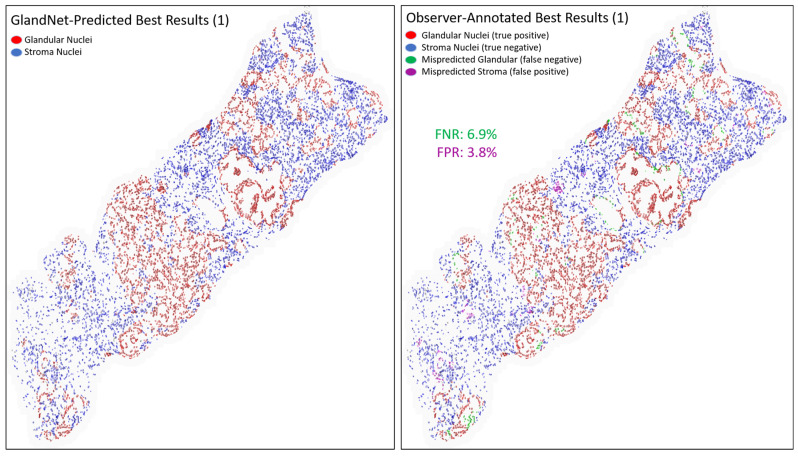
Images for one of the best-predicted 3 biopsies from the final round. GlandNet predictions (**left**) and observer’s annotations (**right**). Red nuclei are correctly identified glandular cells, blue nuclei are correctly identified stroma cells, green nuclei are missed glandular cells, and magenta nuclei are stroma cells incorrectly identified as glandular cells.

**Figure 8:**
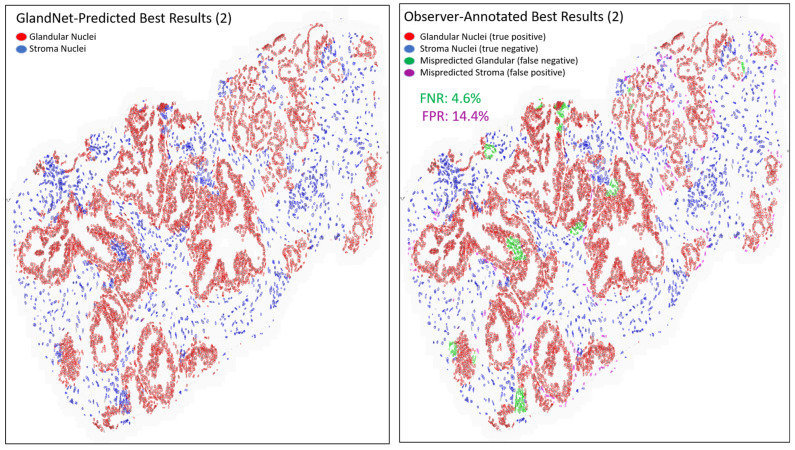
Another image of the best-predicted 3 biopsies from the final round. GlandNet predictions (**left**) and observer’s annotations (**right**). Red nuclei are correctly identified glandular cells, blue nuclei are correctly identified stroma cells, green nuclei are missed glandular cells, and magenta nuclei are stroma cells incorrectly identified as glandular cells.

**Figure 9:**
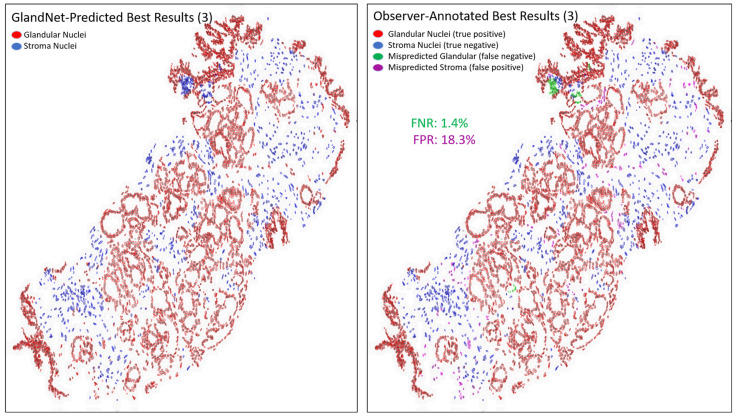
Third images of the best-predicted 3 biopsies from the final round. GlandNet predictions (**left**) and observer’s annotations (**right**). Red nuclei are correctly identified glandular cells, blue nuclei are correctly identified stroma cells, green nuclei are missed glandular cells, and magenta nuclei are stroma cells incorrectly identified as glandular cells.

Table 2 presents the results for the best three predicted biopsies across the hand-annotated 20,735 cells as seen in Figure 7, Figure 8 and Figure 9. The proposed network GlandNet achieved an average prostate glandular cell detection rate of 95.7% (sensitivity), with an F1-score of 95.2%. On the other hand, the stroma cell recognition rate was 87.8% (specificity). The top-ranked three biopsies had an average false positive rate (FPR) of 12.2% and a false negative rate (FNR) of 4.3%. The prostate biopsies in Figure 7 and Figure 9 demonstrated the lowest FPR and FNR values of 3.8% and 1.4%, respectively, demonstrating the effectiveness of GlandNet.

**Figure 10:**
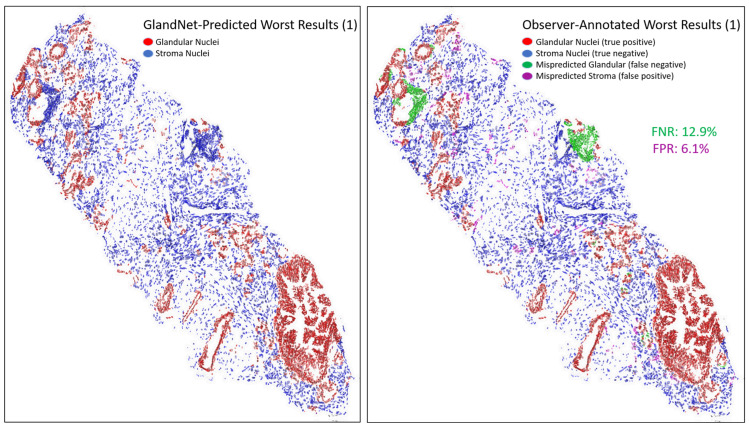
Images for one of the worst-predicted 3 biopsies from the final round. GlandNet predictions (**left**) and observer’s annotations (**right**). Red nuclei are correctly identified glandular cells, blue nuclei are correctly identified stroma cells, green nuclei are missed glandular cells, and magenta nuclei are stroma cells incorrectly identified as glandular cells.

**Figure 11:**
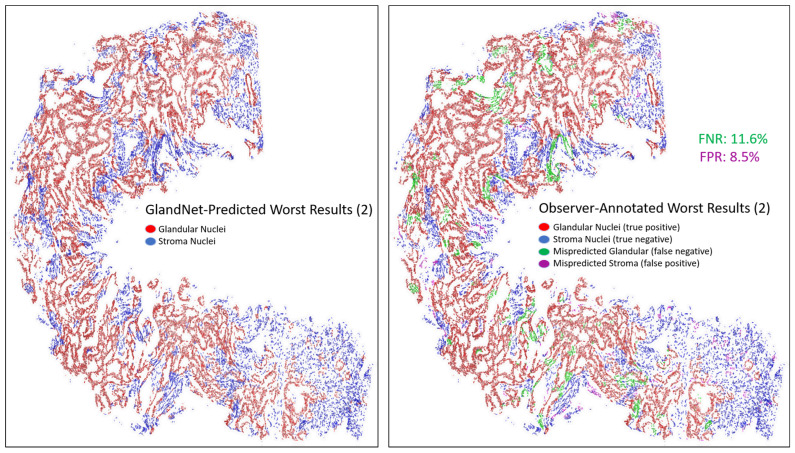
Another image for one of the worst-predicted 3 biopsies from the final round. GlandNet predictions (**left**) and observer’s annotations (**right**). Red nuclei are correctly identified glandular cells, blue nuclei are correctly identified stroma cells, green nuclei are missed glandular cells, and magenta nuclei are stroma cells incorrectly identified as glandular cells.

**Figure 12:**
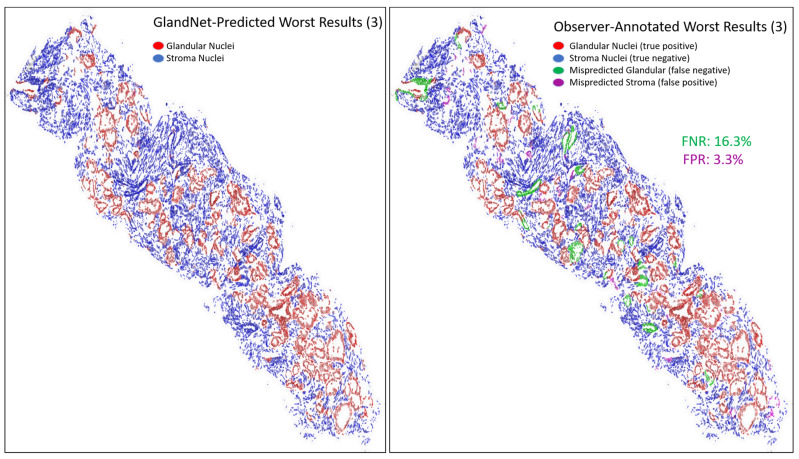
Final images for one of the worst predicted 3 biopsies from the final round. GlandNet predictions (**left**) and observer’s annotations (**right**). Red nuclei are correctly identified glandular cells, blue nuclei are correctly identified stroma cells, green nuclei are missed glandular cells, and magenta nuclei are stroma cells incorrectly identified as glandular cells.

For the visually accessed worst three biopsies (Figure 10, Figure 11 and Figure 12), the overall detection accuracy, specificity, PPV, and PPV were more than 90% whereas the sensitivity, NPV, and F1-scores were smaller (Table 3). The average sensitivity was 86.4%. The GlandNet model had the lowest and highest FNR of 11.6% and 16.3% for misrecognized glandular cells, as highlighted in Figure 11 and Figure 12. Conversely, Figure 11 and Figure 12 displayed the highest and lowest FPR for misrecognized stroma cells for the corresponding needle biopsy images.

**Figure 13:**
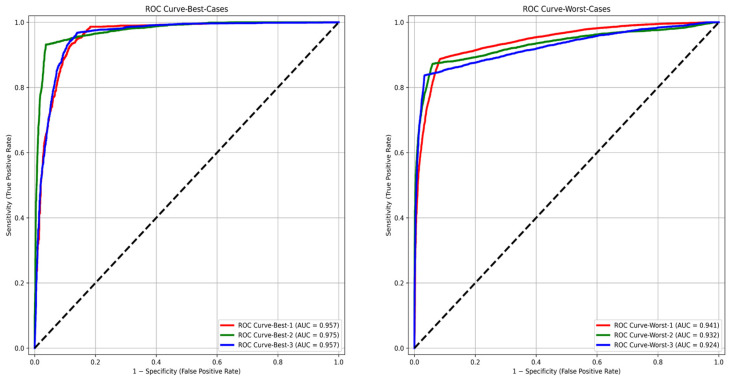
AUC values for ROC curves of best 3 (**left**) and worst 3 (**right**) prediction cases.

**Figure 14:**
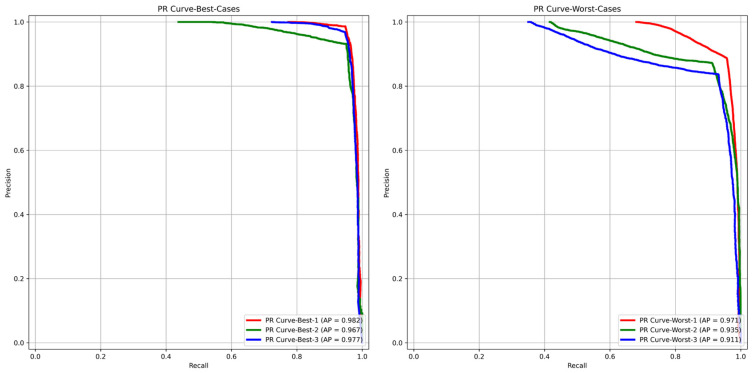
AP values for PR curves of best 3 (**left**) and worst 3 (**right**) prediction cases.

For each of the three best and three worst predicted biopsies, we examined the cell level in the receiver operating characteristic (ROC) curve, which compares TPR (sensitivity) to FPR (1-specificity) for each cell in both the best and worst cases by varying the threshold used on the final classification step; we could fully evaluate how well GlandNet performs. Figure 13 displays GlandNet’s ROC and projected area under the ROC curve (AUC) for the best three biopsies (left) and the worst three biopsies (right). As highly predicted from our observation, the highest AUC of 97.5% was seen for the best scenario, and the lowest AUC of 92.4% was noted for the worst case. Conversely, the average precision (AP) as a function of the threshold used was determined by using the precision (PPV) and recall (sensitivity) of each cell, which is often referred to as the PR curve, as illustrated in Figure 14. In both the ROC and PR analysis, AUC and AP values for the best biopsies were higher than those of the worst cases.

### 3.4. Out-of-Scope Validation: GlandNet Performance on High-Grade Lesion

**Figure 15:**
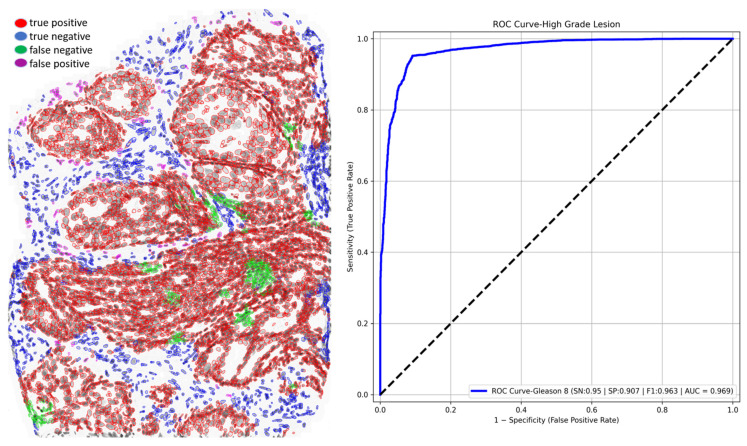
GlandNet performance on a high-grade lesion (Gleason 8). The observer’s annotations vs. prediction results (**left**) and ROC curve (**right**). Red nuclei are correctly identified glandular cells, blue nuclei are correctly identified stroma cells, green nuclei are missed glandular cells, and magenta nuclei are stroma cells incorrectly identified as glandular cells.

Since the biopsy lesions we used for the training, validation, and test sets consisted only of Gleason 6 or 7 cases, GlandNet was applied to a high-grade (Gleason 8) needle biopsy to assess its potential generalizability for Gleason 8 tissue. Figure 15 (left) presents the outcomes obtained by our model, which attained an accuracy of 94.1%, a sensitivity (SN) of 95.0%, a specificity (SP) of 90.7%, a PPV of 97.6%, an F1-score of 96.3%, a FPR of 9.3%, and a FNR of 5.0% for this single instance. Furthermore, GlandNet achieved an AUC of 96.9% on the ROC curve by using the true positive rate and false positive rate of each nucleus. We did not evaluate more instances of high-grade cancers due to data constraints. Our model demonstrates very good generalization in epithelial cell identification, particularly when comparing its performance on high-grade cells to that of earlier low-grade cells.

## 4. Discussion

The pathological hallmark of prostate cancer involves changes in the architecture of the glandular structure within the prostate and changes within the phenotypes of the epithelial cells. The main purpose of developing the GlandNet system was to enhance AI’s ability to assist in the prediction of the biological aggressiveness of prostate cancer, by providing a system that reliably recognizes glandular epithelial cells when they are stained with a DNA-specific stain. Previously [19], we found that, by using nuclear morphology and within nucleus DNA organization in individual cells stained in this fashion to predict biological aggressiveness (response to radiation treatment or predicting active surveillance failure), that knowledge, if the cell was in the stroma or was an epithelial cell in the gland, was beneficial in improving the predictions. While the methodology we use to make the biological aggressiveness predictions can be high throughput, the annotation of the glands is not. Thus, an accurate GlandNet would be very beneficial.

An integration of deep convolutional neural networks, semi-supervised learning, and image data augmentation was trained to recognize epithelial cells. Our observation was that the learning of GlandNet regarding prostatic glands is not adequately generalized in cell-centric Patch-128 patches (Figure 2C). As a consequence of this, the model performed with a relatively low level of sensitivity, with an average FPR of 41.1%. Our approach effectively achieved a balance between the FPR and the FNR while using a nucleus at center of Patches-256 (Figure 2D) as these provided more comprehensive information on the organization of the cells surrounding the cell-centric nucleus (blue box). Notably, we observed that the incorrectly predicted cells (FP and FN) are either in very small glands, are overlapping other nuclei (very nuclei dense areas), or are fuzzy/out of focus (poor quality cells). For example, in Figure 8 and Figure 9 the missed gland cell mistakes are mainly in very small glands or areas of nuclear crowding, and the missed stromal cells are larger nuclei that happen to be arranged in string-like structures. In Figure 10 and Figure 11, this is also true; in addition, missed stromal cells are also mostly small and blurry. Also were the structure of the tissue which appeared much more physically distorted (some glands had a much more stretched-out appearance) as the prediction performance degraded.

Prior AI patch-based deep learning research has mostly focused on the Gleason scoring system without differentiating between epithelial vs. stromal at the individual cell level [10,11,12,13,14]. A few studies used prostate grand segmentation based upon the H&E stain to classify benign (low-grade) and cancerous (high-grade) glands without classifying individual cells [15,16,17]. In contrast, our approach is to use Feulgen-Thionin stains (stoichiometric DNA stain) to effectively recognize and characterize epithelial and stromal cells and nuclei. The majority of previous methods have shown restricted scope when it comes to handling data distributions that were not seen during the training phase. Our proposed semi-supervised training technique showed excellent generalizability on our unseen test cohort.

There are several limitations to our study. Some biopsies saw an increase in the false positive rate (FPR) due to the use of semi-supervised learning approaches. In the top-ranked three scenarios, we observed a maximum false positive rate (FPR) of 18.3%, as seen in the Figure 9. We need to examine these identified nuclei in an independent group of individuals using a well-accepted and agreed-upon reference standard. We have a strong conviction that there was a possibility of error by the human observer in distinguishing between all the gland nuclei in a complicated area of prostate tissue. Furthermore, our model was not trained to distinguish varying Gleason grades inside the prostatic glands, and the biopsies used were all scored as Gleason 6 or 7. However, we pilot-tested our method with a Gleason 8 lesion image as shown in Figure 13. How well GlandNet would perform on additional Gleason 8 or 9 biopsies could differ from the results presented here and requires additional testing on a larger set of Gleason grade 8 or 9 biopsies. Since we were unaware of any studies that combined semi-supervised knowledge with CNN training to detect glandular epithelial cells using the Feulgen-stained biopsies, we were unable to compare GlandNet’s performance to other studies.

One can use immunohistochemistry to identify prostate gland cells; however this is a more costly approach and is not compatible with the Thionin stain used to specifically stain the DNA of the individual cells in the biopsies. Potentially, one could use DAPI (4′,6-diamidino-2-phenylindole) to label the DNA within nuclei and a glandular cell-specific antibody-based immunofluorescence such as a pan cytokeratin (panCK) label to identify glandular cells, but this requires a much more complicated whole slide fluorescence imaging system. We are in the process of developing protocols that make use of immunofluorescence imaging of DAPI and panCK whole slide fluorescence imaging followed by Feulgen-Thionin staining and bright-field imaging of the Thionin stain. Once the two images are aligned, the immunofluorescence image could potentially provide the glandular cell annotation at scale to create a larger annotated (at the individual cell level) gland, a non-gland cohort for further training and validation. However, this method of cell type annotation has its own set of challenges (overlapping of the marker-positive cell cytoplasm of neighboring cells generating false positives in adjacent cells, for example) that need to be addressed before it could be incorporated into a study such as that presented here. In addition, the optimal parameters of GlandNet employed in this study may differ depending on the dataset, as each pathological dataset possesses its own distinctive characteristics.

## 5. Conclusions

GlandNet is an integrated machine-learning network developed to recognize and correctly predict glandular epithelial and non-glandular stromal cells in stoichiometric DNA-stained prostate core needle biopsies with a ~91% to 94% accuracy and a ~90% to 95% F1-score. The model achieved an average true negative rate and average true positive rate of 90.1% and 91% in the test cohort biopsies, with the true negative rate getting as high as 96.7% and the true positive rate getting as high as 98.6% for some biopsies.

The method presented could enhance the ability of AI systems based upon stoichiometric-stained DNA to identify biologically aggressive prostate cancer cells in a higher throughput fashion (vs. human annotation). Implementation of this process could potentially enhance reproducibility and remove human subjectivity in recognizing changes in epithelial tissue that predict biological aggressiveness. Future work will focus on conducting an external validation of GlandNet using additional high-grade Gleason 8 or 9 biopsies to further assess its robustness and generalizability.

## Figures and Tables

**Table 1:** GlandNet’s overall performance when used Patch-128.

Random Splits	Accuracy (%)	Sensitivity (%)	Specificity (%)	PPV (%)	NPV (%)	F1-Score (%)
1	87.6	91.1	84.6	83.3	91.8	87.0
2	88.7	70.3	95.1	83.1	90.3	76.2
3	90.3	15.5	98.3	49.9	91.6	23.6
Average	88.8	58.9	92.6	72.1	91.2	62.3

**Table 2:** Majority vote across the three observers of the best 3 test results archived by GlandNet.

Best 3	Accuracy (%)	Sensitivity (%)	Specificity (%)	PPV (%)	NPV (%)	F1-Score (%)
1	94.9	93.1	96.2	95.1	94.7	94.1
2	92.6	95.3	85.6	94.5	87.5	94.9
3	94.8	98.6	81.8	94.9	94.4	96.7
Average	94.1	95.7	87.8	94.8	92.2	95.2

**Table 3:** Majority vote across three observers of the worst 3 test results archived by GlandNet.

Worst 3	Accuracy (%)	Sensitivity (%)	Specificity (%)	PPV (%)	NPV (%)	F1-Score (%)
1	91.1	87.1	93.9	91.0	91.1	89.0
2	89.4	88.4	91.5	95.7	78.8	91.9
3	92.1	83.7	96.7	93.1	91.7	88.1
Average	90.9	86.4	94.0	93.3	87.2	89.7

## Data Availability

We sought tissue needle biopsy blocks from 82 patients diagnosed with prostate cancer under active monitoring, following the ethical criteria established by the Vancouver Prostate Centre and the University of British Columbia. The signed agreement prohibits us from sharing the data, although any further inquiries about our dataset can be sent to the corresponding authors.

## Supplementary Materials

The following supporting information can be downloaded at https://www.mdpi.com/article/10.3390/cells14100737/s1, Table S1: GlandNet’s best 3 biopsy level validation results after the 1^st^ and 2^nd^ rounds of training; Table S2: GlandNet’s worst 3 biopsy level validation results after the 1^st^ and 2^nd^ rounds of training; Figure S1: GlandNet validation results of the first best-ranked biopsy after the 1^st^ and 2^nd^ rounds of training (red nuclei are correctly identified glandular cells, blue nuclei are correctly identified stroma cells, green nuclei are missed glandular cells, and magenta nuclei are stroma cells incorrectly identified as glandular cells); Figure S2: GlandNet validation results of the third best-ranked biopsy after the 1^st^ and 2^nd^ rounds of training (red nuclei are correctly identified glandular cells, blue nuclei are correctly identified stroma cells, green nuclei are missed glandular cells, and magenta nuclei are stroma cells incorrectly identified as glandular cells); Figure S3: GlandNet validation results of the first worst-ranked biopsy after the 1^st^ and 2^nd^ rounds of training (red nuclei are correctly identified glandular cells, blue nuclei are correctly identified stroma cells, green nuclei are missed glandular cells, and magenta nuclei are stroma cells incorrectly identified as glandular cells); Figure S4: GlandNet validation results of the third best-ranked biopsy after the 1^st^ and 2^nd^ rounds of training (red nuclei are correctly identified glandular cells, blue nuclei are correctly identified stroma cells, green nuclei are missed glandular cells, and magenta nuclei are stroma cells incorrectly identified as glandular cells).

## Abbreviations

The following abbreviations are used in this manuscript:

AI	Artificial intelligence
GlandNet	Glandular cell recognition network
DNA	Deoxyribonucleic Acid
PCa	Prostate Cancer
TME	Tissue Microenvironment
H&E	Hematoxylin and Eosin
MIDI	Musical Instrument Digital Interface
CCD	Charge Coupled Device
ROI	Region of Interest
CMG	Compressed Media Group
CNN	Convolutional Neural Network
VGG16	Deep Learning Architecture
PPV	Positive Prediction Value
NPV	Negative Prediction Value
FPR	False Positive Rate
FNR	False Negative Rate
AUC-ROC	Area Under the Curve-Receiver Operating Characteristic
AP-PR	Average precision (AP) under the precision-recall (PR) curve
panCK	pan Cytokeratin
