# Supplementary material for: Recognizing Epithelial Cells in Prostatic Glands Using Deep Learning
Source: Cells. 2025 May 18;14(10):737. doi: 10.3390/cells14100737 (PMC12109630; doi:10.3390/cells14100737)
Supplement: Supplementary file 1 [file cells-14-00737-s001.zip › cells-3595229-supplementary.pdf]

Supplementary Table S1: GlandNet's best 3 biopsy level validation results after the 1st and 2nd rounds of training

Best 3 biopsies	Results after*	Accuracy (%)	Sensitivity (%)	Specificity (%)	PPV (%)	NPV (%)
1	*round 1st	87.4	85.3	88.0	65.9	95.6
	*round 2nd	83.8	92.2	81.5	57.9	97.4
2	*round 1st	89.5	86.9	91.8	90.7	88.4
	*round 2nd	88.6	94.2	83.5	84.0	94.0
3	*round 1st	91.9	84.4	95.1	88.1	93.4
	*round 2nd	90.2	91.8	89.5	79.0	96.2

Supplementary Table S2: GlandNet's worst 3 biopsy level validation results after the 1st and 2nd rounds of training

Worst 3 biopsies	Results after*	Accuracy (%)	Sensitivity (%)	Specificity (%)	PPV (%)	NPV (%)
1	*round 1st	79.2	82.9	74.8	79.4	78.9
	*round 2nd	76.9	94.1	56.7	71.8	89.2
2	*round 1st	81.2	78.7	84.0	84.5	78.1
	*round 2nd	80.7	92.4	67.7	76.0	89.0
3	*round 1st	85.3	81.5	87.8	81.5	87.8
	*round 2nd	80.7	91.6	73.6	69.5	93.0

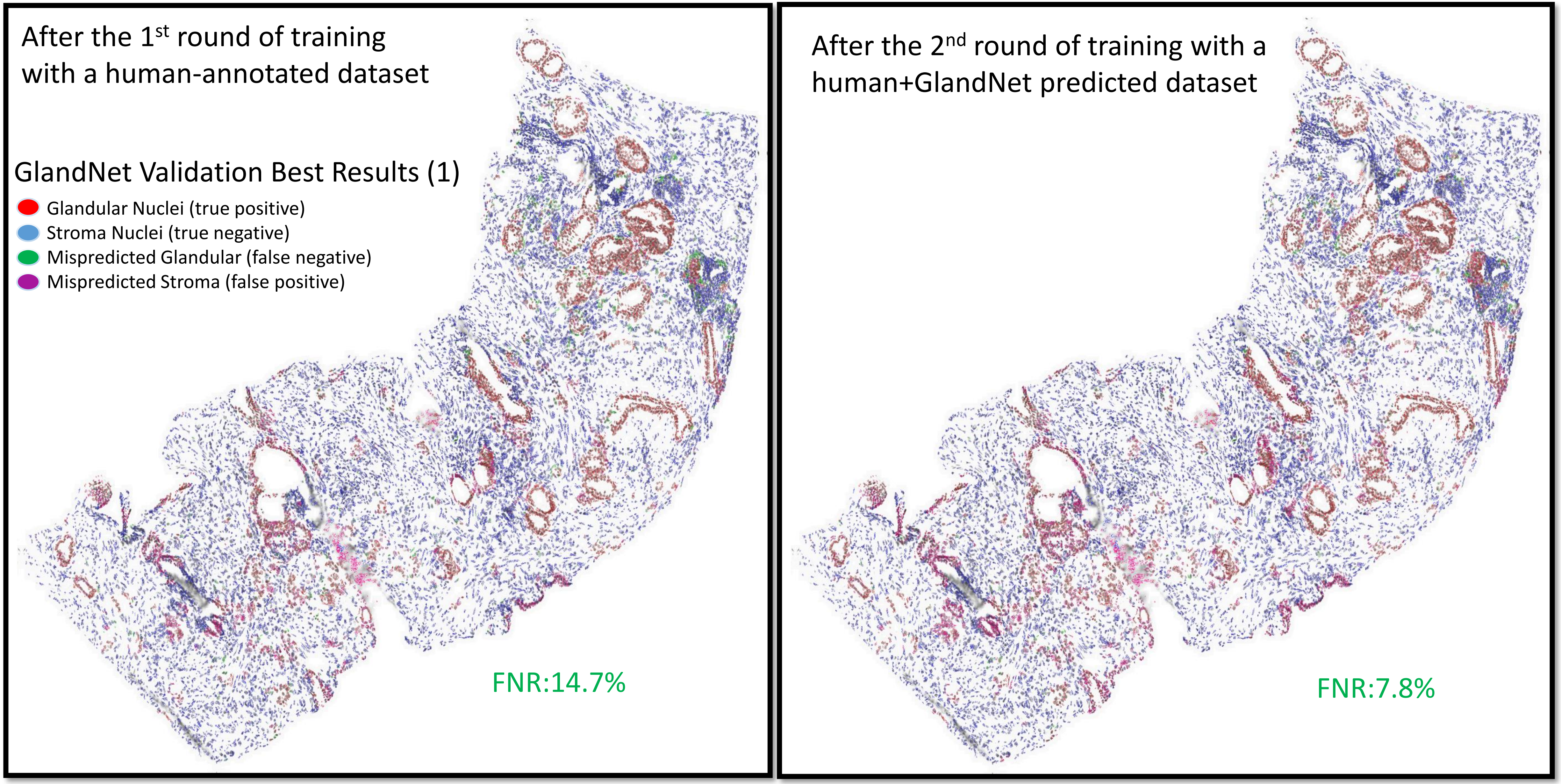

Supplementary Figure S1: GlandNet validation results of the first-best-ranked biopsy after the 1st and 2nd rounds of training (red nuclei are correctly identified glandular cells, blue nuclei are correctly identified stroma cells, green nuclei are missed glandular cells, and magenta nuclei are stroma cells incorrectly identified as glandular cells)

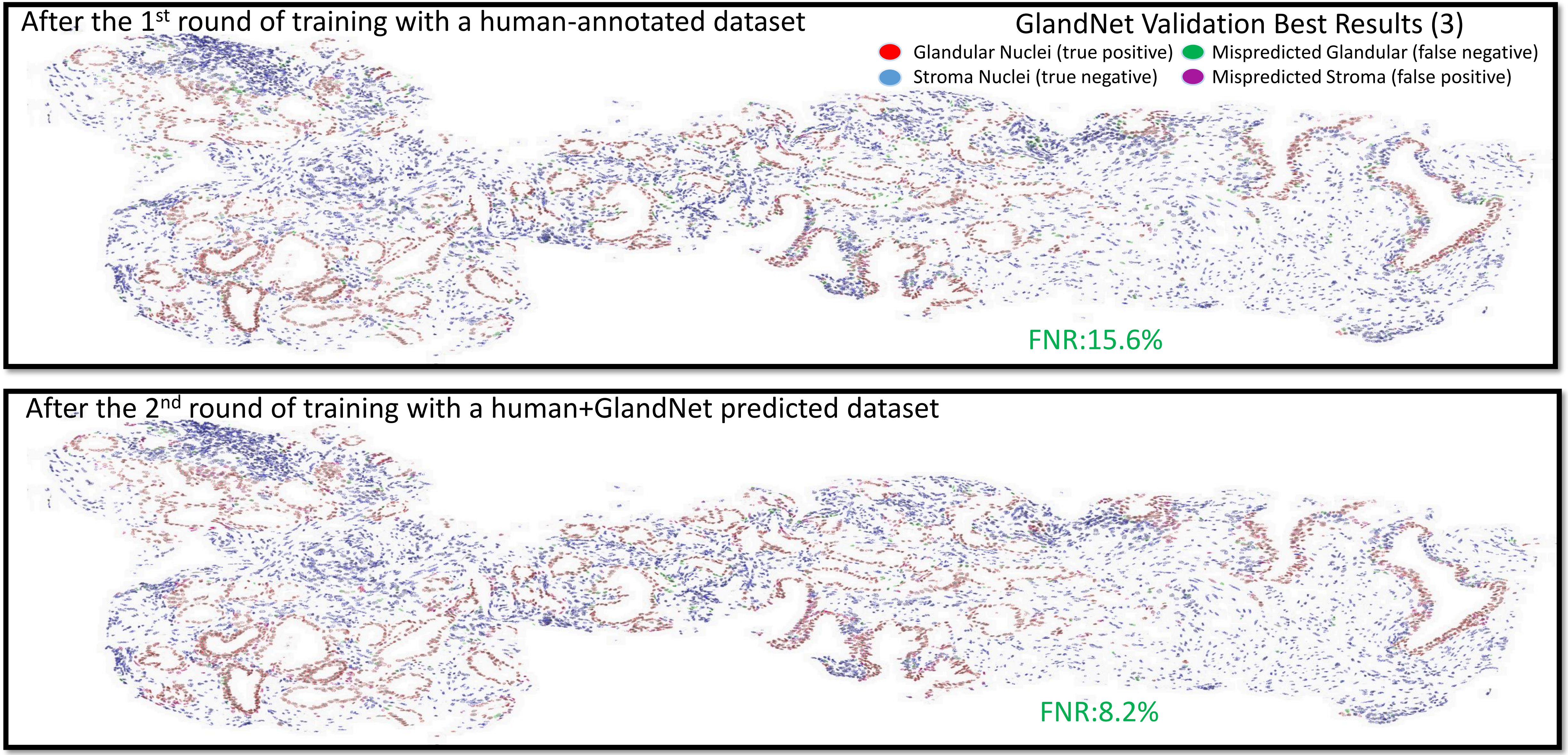

Supplementary Figure S2: GlandNet validation results of the third-best-ranked biopsy after the 1st and 2nd rounds of training (red nuclei are correctly identified glandular cells, blue nuclei are correctly identified stroma cells, green nuclei are missed glandular cells, and magenta nuclei are stroma cells incorrectly identified as glandular cells)

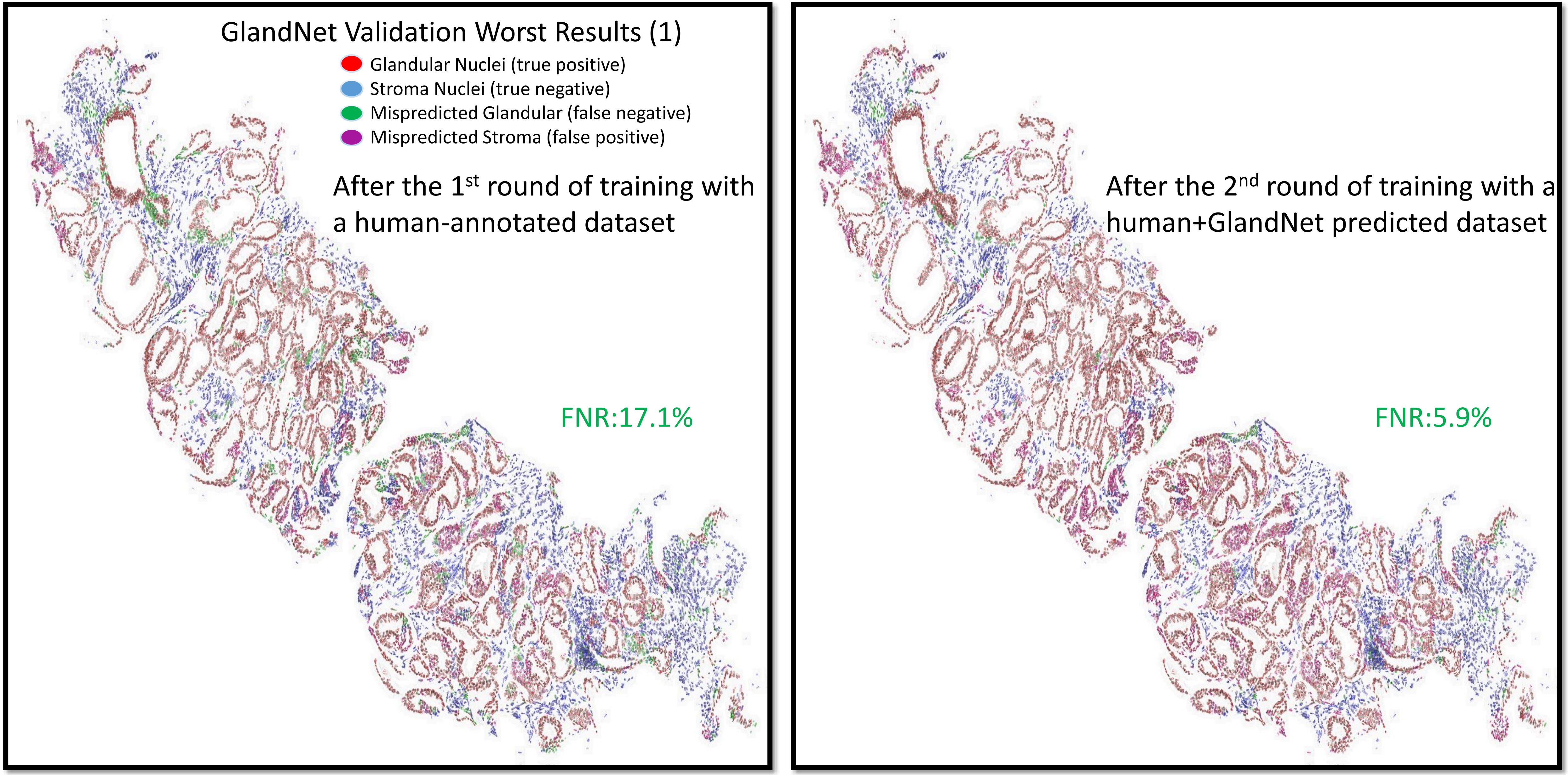

Supplementary Figure S3: GlandNet validation results of the first-worst-ranked biopsy after the 1st and 2nd rounds of training (red nuclei are correctly identified glandular cells, blue nuclei are correctly identified stroma cells, green nuclei are missed glandular cells, and magenta nuclei are stroma cells incorrectly identified as glandular cells)

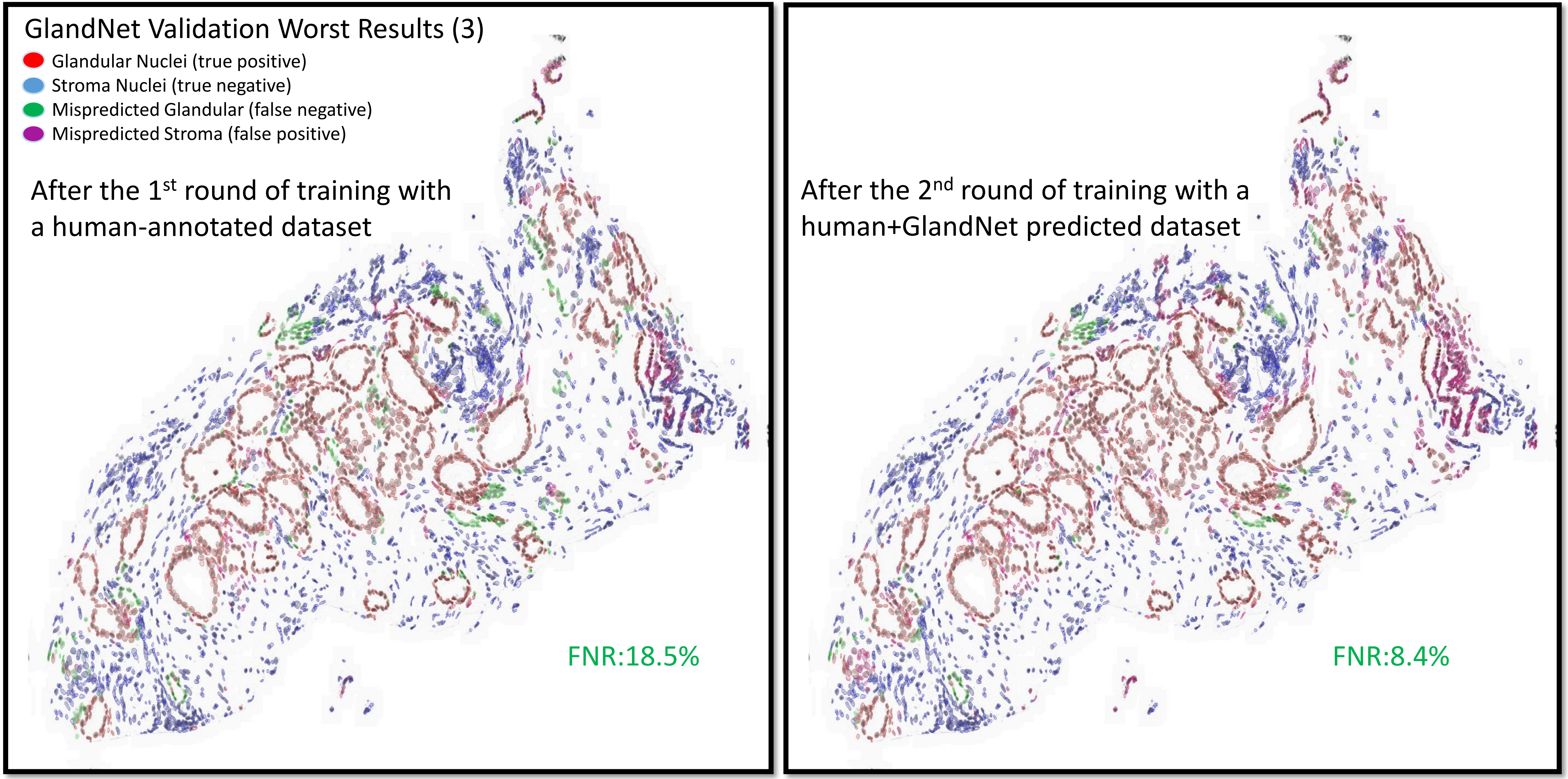

Supplementary Figure S4: GlandNet validation results of the third-best-ranked biopsy after the 1st and 2nd rounds of training (red nuclei are correctly identified glandular cells, blue nuclei are correctly identified stroma cells, green nuclei are missed glandular cells, and magenta nuclei are stroma cells incorrectly identified as glandular cells)
